# Gene network analysis shows immune-signaling and ERK1/2 as novel genetic markers for multiple addiction phenotypes: alcohol, smoking and opioid addiction

**DOI:** 10.1186/s12918-015-0167-x

**Published:** 2015-06-05

**Authors:** Cielito C. Reyes-Gibby, Christine Yuan, Jian Wang, Sai-Ching J. Yeung, Sanjay Shete

**Affiliations:** Department of Emergency Medicine, The University of Texas MD Anderson Cancer Center, Houston, TX 77030 USA; Department of Biostatistics, The University of Texas MD Anderson Cancer Center, Houston, TX 77030 USA

**Keywords:** Pain, Opioid, Smoking, Alcohol, Addiction, Genes, Inflammation, Cancer

## Abstract

**Background:**

Addictions to alcohol and tobacco, known risk factors for cancer, are complex heritable disorders. Addictive behaviors have a bidirectional relationship with pain. We hypothesize that the associations between alcohol, smoking, and opioid addiction observed in cancer patients have a genetic basis. Therefore, using bioinformatics tools, we explored the underlying genetic basis and identified new candidate genes and common biological pathways for smoking, alcohol, and opioid addiction.

**Results:**

Literature search showed 56 genes associated with alcohol, smoking and opioid addiction. Using Core Analysis function in Ingenuity Pathway Analysis software, we found that ERK1/2 was strongly interconnected across all three addiction networks. Genes involved in immune signaling pathways were shown across all three networks. Connect function from IPA My Pathway toolbox showed that DRD2 is the gene common to both the list of genetic variations associated with all three addiction phenotypes and the components of the brain neuronal signaling network involved in substance addiction. The top canonical pathways associated with the 56 genes were: 1) calcium signaling, 2) GPCR signaling, 3) cAMP-mediated signaling, 4) GABA receptor signaling, and 5) G-alpha i signaling.

**Conlusions:**

Cancer patients are often prescribed opioids for cancer pain thus increasing their risk for opioid abuse and addiction. Our findings provide candidate genes and biological pathways underlying addiction phenotypes, which may be future targets for treatment of addiction. Further study of the variations of the candidate genes could allow physicians to make more informed decisions when treating cancer pain with opioid analgesics.

**Electronic supplementary material:**

The online version of this article (doi:10.1186/s12918-015-0167-x) contains supplementary material, which is available to authorized users.

## Background

Pain is a debilitating problem that cancer patients face, impairing their quality of life. Pain may be related to multiple factors, including radiotherapy, chemotherapy, surgery, and cancer progression. In order to mitigate therapy-related pain or cancer-related pain, physicians often prescribe opioid analgesics to cancer patients [1, 2]. The prescription of opioids for pain carries risk for opioid abuse and addiction. Because of the increased survival rate in cancer patients, their exposure to prescriptions of opioids are also prolonged, further increasing their risk for opioid abuse and addiction [3–5].

Studies showed that opioid abuse was associated with past histories of drug and alcohol abuse in patients treated for cancer-related pain with opioid analgesics [6, 7]. Several clinical trials also found that patients with a history of cigarette smoking and illicit drug abuse had a significantly higher risk for opioid addiction than those without the history [8–11]. Taken together, these studies suggest that past addictive behaviors to various substances may predict opioid addiction in cancer patients with opioid prescriptions for pain. However, very few studies have explored whether there exists a genetic basis and common pathways to the relationship between smoking, alcohol, and opioid addiction.

Bioinformatics uses methods and software tools to organize and analyze biological data [12]. Specifically, gene network analyses have been used frequently to identify genes associated with drug abuse and addiction [13–15]. However, there has been limited application of bioinformatics in understanding multiple addiction phenotypes, specifically, smoking, alcohol and opioid addiction. We hypothesize that the associations between alcohol, smoking, and opioid addiction observed in the clinical setting have a genetic basis.

The goal of the current study is to use bioinformatics tools to determine whether there exists a genetic basis and common pathways to the relationship between smoking, alcohol, and opioid addiction and identify new candidate target genes. Understanding the genetic bases of addiction will underscore the importance of integrating genetic studies into the process of drug administration, as well as allow clinicians to more accurately tailor a patient’s drugs and dosage based on medical history and genetic risk factors [16].

## Methods

With the goal of identifying commonly shared genes for alcohol, smoking and opioid addiction we conducted a literature search as described below. Subsequently, using genes pooled from literature as a starting point, we performed gene network analyses: a) specific to each phenotype (Phenotype Specific Biological Network) and b) commonly shared between alcohol, smoking and opioid addiction (Common Biological Network). Finally, we used the Connect function from IPA My Pathway toolbox to connect the commonly shared genes of the three phenotypes to the signaling network involved in neuronal adaptation/plasticity in substance addiction [17, 18].

### Literature search

**Fig. 1:**
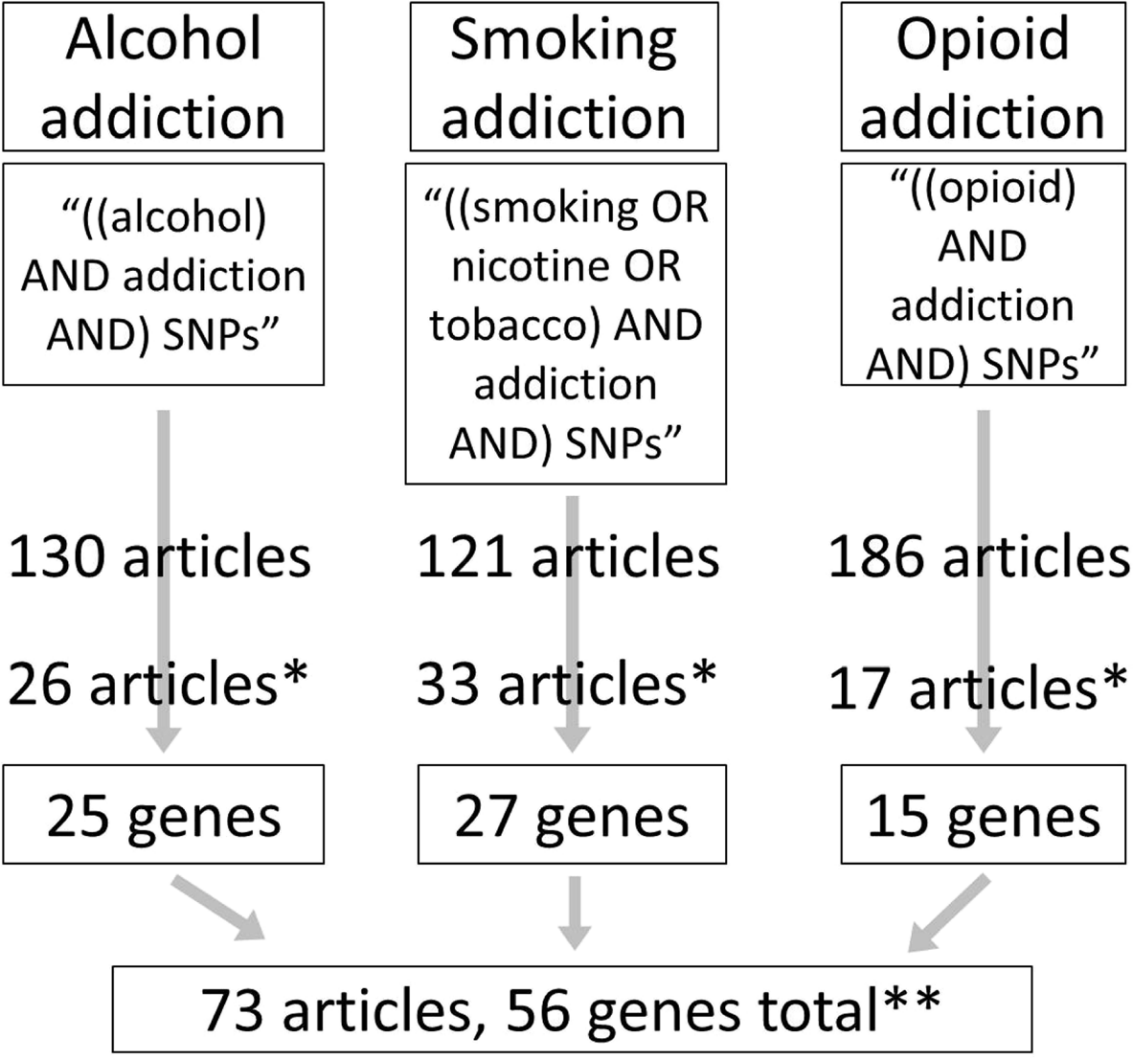
Literature search flowchart. *Subset after using the following Exclusion criteria: Literature review/meta-analysis, non-human experiments, other mental disorders, recovery/withdrawal, unrelated to phenotype, genes that were not replicated in or confirmed by at least one independent study. **Some overlaps between phenotypes for articles and genes

Each substance of abuse was searched on the PubMed database using the keywords “addiction” and “SNPs” in July 2014. Specifically, we used the term “alcohol addiction SNPs” for alcohol addiction, “smoking/nicotine/tobacco addiction SNPs” for smoking addiction, and “opioid addiction SNPs” for opioid addiction (Fig. 1). No limitations were placed on the year of publication. Non-human trials, literature reviews, and meta-analyses were excluded. Articles about treatment of drug addiction and drug addiction in patients with mental illnesses were also excluded. The genes reported in the literature to be statistically significantly associated with one of the addiction phenotypes were included in the pathway analysis and are called focus genes. The genes that were not replicated in an independent study were excluded. Figure 1 shows the criteria of the literature search.

### Ingenuity pathway analysis

Ingenuity Pathway Analysis (IPA) was used to produce a comprehensive analysis of the genes commonly shared in these addiction pathways. IPA is a software used to connect molecules based on the Ingenuity Knowledge Base, its database of information on biomolecules and their relationships [19]. The Core Analysis function was used to compare genes pooled from literature for each phenotype of addiction with the genes and other molecules in IPA’s database and generates gene networks based on their interactions.

**Fig. 2:**
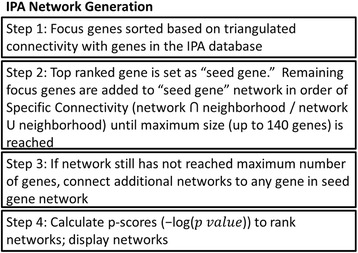
IPA network generation process

We first designated a set of criteria for the molecules included in the Core Analysis. The following criteria were used: genes and endogenous chemicals, maximum molecules per network (140) and networks per analysis (25), humans, tissues and primary cells. Figure 2 illustrates the steps of the network generation process [20]. The resulting networks are then scored based on the negative base 10 logarithm of the p-value obtained using the Fisher’s exact test (i.e., -log_10_(p-value)), with the null hypothesis being that the molecules within the networks were connected based on chance.

### Phenotype specific biological network

Gene networks were created for each addiction phenotype. Only the networks with a p-score of 5 or higher were considered significant (i.e., p-value ≤ 10^−5^), a nominal significance used in previous studies [21]. The genes in each network were ranked based on number of edges, or interactions with other genes in the network.

### Common biological network

**Fig. 3:**
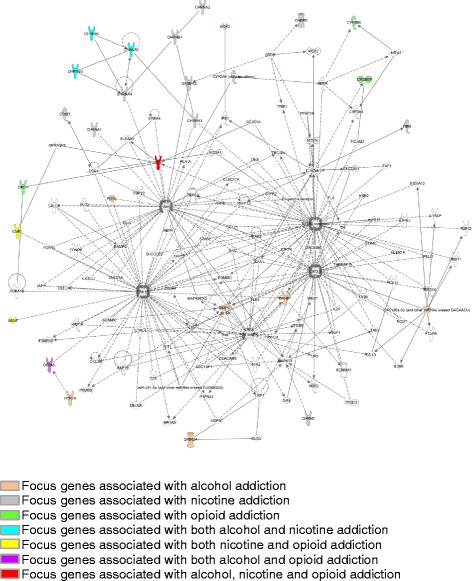
Network generated by pooling all 56 focus genes for alcohol, nicotine and opioid addiction (p-score = 45)

In order to identify the shared genes, the opioid addiction network was compared with alcohol and smoking addiction networks. In addition, another network was generated by combining all 56 focus genes for all three addiction phenotypes (Fig. 3). In these analyses, only the network with a score ≥ 5 was considered significant [21]. Supplementary to the gene network, IPA also provides a list of top canonical pathways associated with the focus genes, along with a Fisher’s exact test p-value and the ratio between the number of focus genes in the canonical pathway and the total number of molecules in the canonical pathway. In this study, we also reported the top canonical pathways associated with all 56 focus genes for all three addiction phenotypes.

Finally, in order to understand the biological context of the gene network (association of genetic variations with addiction to opioids, alcohol and nicotine), we used the Connect function from IPA My Pathway toolbox to connect the commonly shared genes of three phenotypes to the signaling network involved in neuronal adaptation/plasticity in substance addiction [17, 18]. The Connect function adds specific interactions between molecules. While performing this analysis, we limited the interactions from only human studies. All results were generated through the use of Ingenuity® iReport [19].

## Results

### Literature search

A total of 73 unique articles were extracted based on the PubMed search for a thorough review. Figure 1 illustrates how the PubMed search produced this final list of articles for literature review. The articles associated with the corresponding type of addiction were summarized in tabular format (Tables 1, 2, 3). This resulted in a list of 56 focus genes total (Fig. 1), and each of these genes was used in the IPA Core Analysis. Opioid receptor genes [22] were frequently studied for alcohol and opioid addiction [22–28]. Nicotinic acetylcholine receptor genes were widely explored for alcohol and nicotine addiction [29–46]. Dopamine receptor genes were frequently explored in all three phenotypes [5, 27, 47–53]. Several overlapping focus genes across the three addiction phenotypes were observed, including DRD2 and CRHR1 for all three phenotypes, OPRM1 for alcohol and opioid addiction network, and BDNF and CNR1 for nicotine and opioid addiction network (Table 4). The 56 focus genes were subsequently used as seed genes in Ingenuity Pathway Analysis.

**Table 1:** Summary of literature search - alcohol addiction

Author	Ethnicity	Sample size	Phenotype	Salient gene(s)	Salient SNP(s)	Statistical analysis
Batel et al. [47]	EA	134	Alcohol dependence	DRD1	rs686	P = 0.0008
Bierut et al. [77]	EA, AA	5632	Increased aversion from alcohol	ADH1B	rs1229984	OR = 0.34 P = 6.6E-10
Cao et al. [78]	Han Chinese	603	Alcohol addiction	5-HTR	rs6313	OR = 0.71 P = 0.001
Chen et al. [79]	EA, AA	3627	Alcohol addiction	PKNOX2	rs1426153 rs11220015 rs11602925 rs750338 rs12273605 rs10893365 rs10893366 rs12284594	P = 5.75E-5, 6.86E-5, 4.24E-5, 4.26E-5, 3.0E-4, 1.72E-5, 1.37E-5, 1.97E-6
Deb et al. [25]	South Asian	144	Alcohol addiction	OPRM1	rs1799971	P = 0.02
Desrivieres et al. [80]	E	145	Drinking behavior	P13K	rs2302975 rs1043526	P = 0.0019, 0.0379
Enoch et al. [81]	AA	360	Alcohol addiction	HTR3B	rs1176744	P = 0.002
Ehringer et al. [35]	EA, Hisp, AA	108	Alcohol response	CHRNB2	rs2072658	
Haller et al. [37]	EA, AA	1315	Alcohol addiction	CHRNB3	rs149775276	P = 2.6E-4 for EA, P = 0.006 for AA
Hill et al. [82]	EA	1000	Alcohol dependence	KIAA0040	rs2269650 rs2861158 rs1008459 rs2272785 rs10912899 rs3753555	P = 0.033, 0.037, 0.014, 0.062, 0.035, 0.020
Kalsi et al. [83]	EA, AA	847	Alcohol addiction	DKK2	rs427983 rs419558 rs399087	P = 0.007
Kumar et al. [26]	Bengali/Hindu	310	Alcohol addiction	OPRM1	rs16918875 rs702764 rs963549	P = 0.0364
Kuo et al. [84]	E	1238	Initial sensitivity to alcohol	GAD		P = 0.002
London et al. [85]	EA		Risk for alcohol addiction	ANKK1	rs1800497	P = 0.001
Mignini et al. [51]	E	560	Dopaminergic system; alcohol dependence	DRD2/ANKK1	rs1800497	P = 0.023
Munoz et al. [86]	E	1533	Number of drinks per day	ADH1B, ADH6	rs1229984 in ADH1B rs3857224 in ADH6	rs1229984: OR = 0.19, P = 4.77E-10 for men, OR = 0.48, P = 0.0067 for women; rs38572: OR = 1.61, P = 1.01E-3 for women, NS for men
Novo-Veleiro et al. [87]	E	457	Risk for alcohol addiction	miR-146a	rs2910164	OR = 1.615 P = 0.023
Preuss et al. [88]	E (German & Polish)	3091	Alcoholism	ADH4	rs1800759 rs1042364	rs1800759: OR = 0.88 rs1042364: OR = 0.87
Ray et al. [89]	CA, As, Latino, NA, AA	124	Level of response to alcohol/drinking problems	GABRG1	rs1497571	P < 0.01
Samochowiec et al. [90]	EA	275	Alcohol dependence	MMP-9	rs3918242	P < 0.01
Schumann et al. [91]	E	1544	Alcohol dependence	NR2A, MGLUR		OR = 2.35, 1.69
Treutlein et al. [92]	E	296	Potential alcohol dependence	CRHR1		
Wang et al. [42]	EA, AA	2309	Alcohol dependence	CHRNA5	rs680244	P = 0.003
Wang et al. [93]	EA	2010	Alcohol dependence	C15orf53	rs12903120 rs12912251	rs12903120: P = 5.45E − 8
Xuei et al. [94]	EA	1923	Risk for alcohol addiction	GABRR1, GABRR2	rs17504587 rs282129 rs13211104 rs9451191 rs2821211 rs6942204	P = 0.04. 0.03, 0.03, 0.021, 0.025, 0.04
Yang et al. [95]	EA, AA	3564	Alcohol dependence	HTR3B	rs3891484 rs375898	D’ > 7

**Table 2:** Summary of literature search - smoking addiction

Author	Ethnicity	Sample size	Phenotype	Salient gene(s)	Salient SNP(s)	Statistical analysis
Agrawal et al. [96]	EA	1929	Nicotine dependence	GABRA4, GABRA2, GABRE		P = 0.030
Agrawal et al. [97]	EA	1921	Nicotine dependence	GABRA4, GABRA2		P = 0.002
Anney et al. [98]	E	815	Cigarette dose	CHRM5	rs7162140	P = 0.01
Baker et al. [31]	EA	886	Nicotine dependence	CHRNA5-A3-B4		P = 0.04
Berrettini et al. [99]	EA	1276	Nicotine addiction	CYP2A6	rs410514431	P = 1.0E-12
Beuten et al. [100]	EA, AA	2037	Nicotine dependence	BDNF	rs6484320 rs988748 rs2030324 rs7934165	P = 0.002
Beuten et al. [101]	EA, AA		Nicotine dependence	GABAB2	rs2491397 rs2184026 rs3750344 rs1435252 rs378042 rs2779562 rs3750344	P = 0.003
Beuten et al. [102]	EA, AA		Nicotine dependence	COMT	rs933271 rs4680 rs174699	P = 0.0005
Broms et al. [32]	E	1428	Nicotine dependence	CHRNA5, CHRNA3, CHRNB4	rs2036527 rs578776 rs11636753 rs11634351 rs1948 rs2036527	P = 0.000009, 0.0001, 0.0059, 0.0069, 0.0071, 0.0003
Chen et al. [103]		688	Nicotine dependence	CNR1	rs2023239 rs12720071 rs806368	P < 0.001
Chen et al. [79]	EA, AA	3627	Nicotine addiction	PKNOX2	rs1426153 rs11220015 rs11602925 rs750338 rs12273605 rs10893365 rs10893366 rs12284594	P = 0.0159, 0.0163, 0.0136, 0.0491, 0.0921, 0.0411, 0.0621, 0.0239
Conlon et al. [33]	EA	1122	Nicotine dependence	CHRNA5, CHRNA3, AGPHD1	rs16969968 rs578776 rs8034191	OR = 3.2, 2.8, 0.3
Culverhouse et al. [34]	AA, EA	18500	Nicotine dependence	CHRNB3, CHRNA7	rs13273442	P = 0.00058 for EA, 0.05 for AA
Docampo et al. [104]	E	752	Lower risk for smoking behavior	NRXN3	rs1424850 rs221497 rs221473	rs1424850: OR = 0.55, P = 0.0002
						rs221497: OR = 0.47, P = 0.0020
						rs221473: OR = 0.54, P = 0.0009
Ehringer et al. [35]	EA, Hisp, AA	108	Nicotine response	CHRNB2	rs2072658	
Ella et al. [105]	Japanese	2521	Nicotine addiction	DBH	rs5320	P = 0.030
Gabrielsen et al. [36]	Norwegian	155941	Smoking status(cigarettes per day, duration, packs per year)	CHRNA5/A3/B4	rs16969968	P = 3.15E-25, 1.11E-6, 3.01E-23 (respectively for phenotypes)
Huang et al. [106]	EA, AA	3403	Nicotine dependence	ANKK1	rs2734849	P = 0.0026
Lang et al. [107]	E	320	Smoking behavior	BDNF		P = 0.045
Li et al. [38]	EA, AA	2037	Nicotine dependence	CHRNA4	rs2273504 rs1044396 rs3787137 rs2236196	
Liu et al. [108]	EA, AA	2091	Smoking behavior	IL15	rs4956302	P = 8.8E-8
Ma et al. [109]	EA, AA	2037	Nicotine dependence	DDC	rs3735273 rs1451371 rs3757472 rs3735273 rs1451371 rs2060762	P = 0.005, 0.006
Mobascher et al. [110]	German	5500	smoking behavior/nicotine addiction	CHRM2	rs324650	OR = 1.17
Nees et al. [39]	E, EA	965	Nicotine dependence	CHRNA5/A3/B4	rs578776	P < 0.05
Sherva et al. [40]	EA, AA	435	Smoking	CHRNA5	rs16969968	P = 0.0001
Rice et al. [29]	EA, AA	3365	Nicotine dependence	CHRNB3	rs1451240	P = 2.4E-8
Sarginson et al. [30]	EA, Asian, AA, Hispanic	577	Smoking behavior	CHRNA5/A3/B4	rs16969968 rs1051730	P < 0.0001
Sorice et al. [41]	E	2272	Smoking behavior	CHRNA5-A3-B4	rs1051730	P = 0.0151, 0.022, 0.22 for three populations
Voisey et al. [52]	EA	378	Nicotine dependence	DRD2	rs1800497	P = 0.0003
Wang et al. [43]	EA, AA	3622	ND (smoking quantity and FTND)	CHRNA2, CHRNA6	EA: rs3735757 rs2472553	EA: P = 0.0068 for FTND, AA: P = 0.0043 for SQ and 0.00086 for FTND
Wassenaar et al. [44]	E	860	Nicotine dependence	CYP2A6 and CHRNA5-A3-B4	rs1051730	P =0.036
Weiss et al. [45]	E	2827	Nicotine dependence	CHRNA5-A3-B4	rs17486278	P = 0.0005
Zeiger et al. [46]	EA, Hisp	1056	Response to smoking	CHRNA6, CHRNB3	rs4950 rs13280604 rs2304297	P = 0.043, 0.011, 0.053

**Table 3:** Summary of literature search - opioid addiction

Author	Ethnicity	Sample size	Phenotype	Salient gene(s)	Salient SNP(s)	Statistical analysis
Beer et al. [22]	E	284	Opioid dependence	GAL, OPRD1	rs948854 rs2236861	P = 0.001
Bunten et al. [23]		184	Opioid addiction	OPRM1	rs1799971	P = 0.0046
Compton et al. [24]	EA	109	Opioid addiction	OPRM1	rs1799971	
Clarke et al. [111]	Han Chinese	858	Opioid dependence	PDYN	rs1997794 rs1022563	P = 0.019, 0.006
Clarke et al. [48]	EA, AA	992	Opioid addiction	DRD2	rs1076560	OR = 1.29, P = 0.0038
Crist et al. [112]	EA, AA	671	Opioid addiction	WLS	rs3748705 (AA) rs983034 rs1036066 (EA)	AA: P = 0.025EA: P = 0.043, 0.045
de Cid et al. [113]	E	91	Opioid Addiction	BDNF		
Doehring et al. [49]	CA	184	Opioid addiction	DRD2	rs1076560 rs1799978 rs6277 rs12364283 rs1799732 rs6468317 rs6275 rs1800498 rs1800497	P = 0.022, 0.048
Gelernter et al. [114]	EA, AA	8246	Opioid dependence	KCNG2	rs62103177	P = 3.60E-10
Herman et al. [115]	EA, AA	1367	Opioid dependence	CNR1	rs6928499 rs806379 rs1535255 rs2023239	
Ho et al. [50]	Chinese	252	Opioid dependence	DRD4		P = 0.041
Kumar et al. [116]	South Asian	260	Opioid dependence	CREBBP	rs3025684	P < 0.0001
Kumar et al. [26]	Bengali/Hindu	330	Opioid addiction	OPRM1	rs16918875 rs702764 rs963549	P = 0.0264
Levran et al. [117]		74	Opioid addiction	CYP2B6		
Liu et al. [118]	African	3627	Opioid addiction	NCK2	rs2377339	P = 1.33E-11
Nagaya et al. [28]	Asian	160	Opioid addiction	OPRM1	rs1799972	OR = 1.77, P < 0.0001
Zhu et al. [53]	Chinese	939	Opioid dependence/addiction	DRD1	rs686	P = 0.0003

**Table 4:** Overlapping genes for networks of nicotine, alcohol and opioid addiction; focus genes from literature are bolded

A: Opioids ∩ Alcohol		B: Opioids ∩ Nicotine		C: Opioids ∩ Alcohol ∩ Nicotine	
Molecule	Edges in opioid network/edges in alcohol network	Molecule	Edges in opioid network/edges in nicotine network	Molecule	Edges in opioid network/edges in alcohol network/edges in nicotine network
NFkB (complex)	112/86	ERK1/2	74/76	ERK1/2	74/62/76
ERK1/2	74/62	ARRB2	8/3	**DRD2**	**6/3/4**
IL1R1	7/4	**DRD2**	**6/4**	TAP1	5/5/3
IL1	6/8	HSPD1	5/4	SAA	4/3/4
DEFB4A/DEFB4B	6/4	TAP1	5/3	PSMB9	4/3/3
**DRD2**	**6/3**	SAA	4/4	TAPBP	4/3/3
ELANE	5/6	PSMB9	4/3	ELF3	4/3/2
F2RL1	5/6	TAPBP	4/3	TAC1	4/3/2
TAP1	5/5	ELF3	4/2	CLEC11A	3/4/2
F2R	5/3	TAC1	4/2	SMPD2	3/3/3
ADRBK1	5/2	PSMB10	3/3	CXCL3	3/3/2
Ikb	4/4	SMPD2	3/3	P2RY6	3/3/2
CXCL2	4/3	AKAP13	3/2	PSMB10	3/2/3
ELF3	4/3	CLEC11A	3/2	AKAP13	3/2/2
FPR2	4/3	CXCL3	3/2	TLR6	3/2/2
PSMB9	4/3	P2RY6	3/2	**CRHR1**	**2/4/3**
SAA	4/3	TLR6	3/2	CD244	2/3/3
TAC1	4/3	CD244	2/3	CXCL5	2/3/2
TAPBP	4/3	**CRHR1**	**2/3**	CCL21	2/2/2
DEFB103A/DEFB103B	4/2	CCL21	2/2	GMFG	2/2/2
LTF	3/5	**CNR1**	**2/2**		
TNFSF11	3/5	CXCL5	2/2		
TNFSF15	3/5	GMFG	2/2		
CLEC11A	3/4	GPRASP1	2/2		
TLR1	3/4	**BDNF**	**2/1**		
CXCL3	3/3				
KLF6	3/3				
P2RY6	3/3				
SMPD2	3/3				
AKAP13	3/2				
ARF6	3/2				
IER3	3/2				
PSMB10	3/2				
TLR6	3/2				
TRPC6	3/2				
**CRHR1**	**2/4**				
CCL22	2/3				
CD244	2/3				
CXCL5	2/3				
CC2D1A	2/2				
CCL21	2/2				
GMFG	2/2				
SH3GLB2	2/2				
STAB2	2/2				
TSC22D3	2/2				
**OPRM1**	**1/2**				

### IPA – Phenotype-specific biological network

Individual gene networks were generated through IPA’s Core Analysis for each addiction phenotype (Additional file 1: Figures S1-S3). TNF, NF-κB, and ERK1/2 were present as highly interconnected genes for alcohol addiction (103, 86, and 62 edges, respectively). For nicotine addiction, TNF, ERK1/2 and Akt had the most edges (85, 76, and 53, respectively). NF-κB, RELA, and ERK1/2 were most interconnected for opioid addiction (112, 92, and 74 edges respectively).

### IPA – Common biological network

Table 4 lists overlapping genes for alcohol and opioids (A), smoking and opioids (B), and all three addiction phenotypes (C). Genes were ranked by the number of edges within the opioid network. The network for opioid addiction was found to have the most number of genes that overlap with the network for alcohol addiction relative to the smoking addiction genes. ERK1/2 was found to be very strongly interconnected across all three addiction networks with 74 edges in opioid network, 62 edges in alcohol network and 76 edges in nicotine network (Table 4, panel C). ERK1/2 also shows with highest number of edges in opioid and nicotine network (Table 4, panel B) and second highest edges in opioid and alcohol network (Table 4, panel A). We also noticed that some commonly shared genes are involved in the immune response. Specifically, the immune response genes that were common in the three networks (panel C) were: corticotropin-releasing hormone receptor 1 (CRHR1), chemokine ligand 21 (CCL21), chemokine ligand 3 (CXCL3), chemokine ligand 5 (CXCL5) and toll-like receptor 6 (TLR6). In addition to the above genes, the following immune response genes were also found in opioid and alcohol genes networks (panel A): beta-defensin 103 (DEFB103A/DEFB103B), beta-defensin 2 (DEFB4A/DEFB4B), elastase neutrophil expressed (ELANE), protease activated receptor 2 (F2RL1), lactoferrin (LTF), nuclear factor kappa-light-chain-enhancer of activated B cells (NF-kappa B), toll-like receptor 1 (TLR1), TSC22 domain family protein 3 (TSC22D3), chemokine ligand 22 (CCL22), chemokine ligand 2 (CXCL2), interleukin 1 receptor type 1 (IL1R1), tumor necrosis factor ligand superfamily member 11 and 15 (TNFSF11 and TNFSF15).

**Fig. 4:**
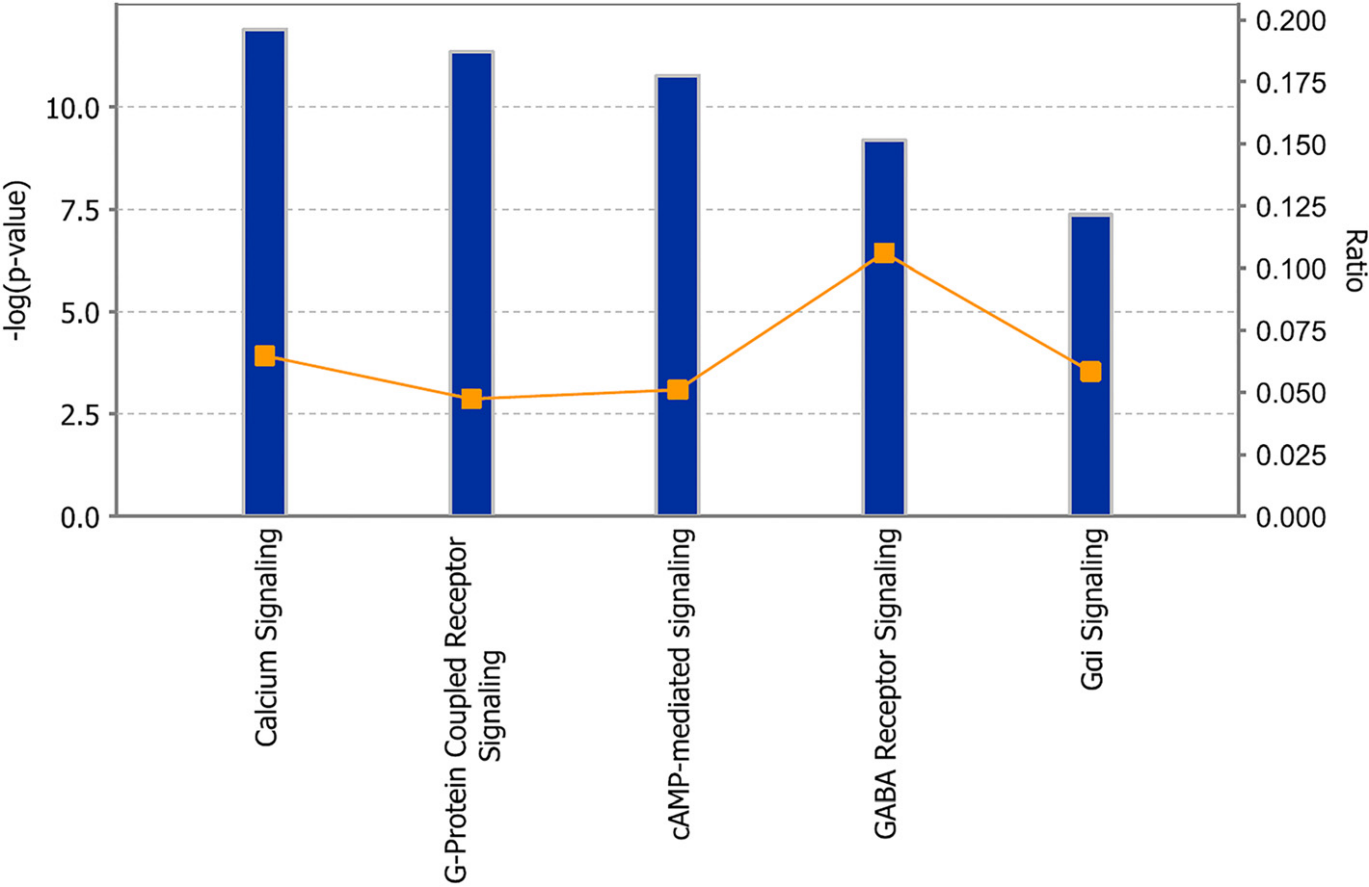
Top canonical pathways obtained by pooling all 56 focus genes for alcohol, nicotine and opioid addiction. Blue bars: p-score for each of the canonical pathways. Yellow lines: ratio for each of the canonical pathways, calculated as the number of focus genes included in the canonical pathway divided by the total number of genes that constitute the canonical pathway

By pooling all 56 focus genes from three addiction phenotypes, a total of 8 networks were generated by using IPA Core Analysis. Figure 3 shows the network with the highest statistical significance (p-value = 10^−45^). Figure 4 shows the top canonical pathways for the combined focus genes, including calcium signaling, GPCR signaling, cAMP-mediated signaling, GABA receptor signaling, and Gαi signaling (p-values = 1.26E-12, 4.45E-12, 1.71E-11, 6.3E-10, 4.29E-8).

### Biological context

**Fig. 5:**
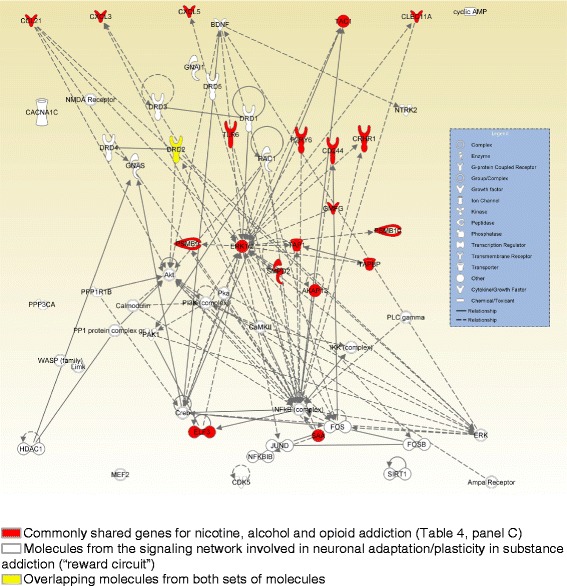
The links of genes associated with addiction to opioids, tobacco and alcohol to components of the brain “reward circuit”

Finally, we used the “Connect” function from IPA My Pathway toolbox to connect the commonly shared genes (i.e., overlapping genes) related to addiction to opioids, alcohol and nicotine (Table 4, panel C) to the signaling network involved in neuronal adaptation/plasticity in substance addiction (Fig. 5) [17, 18]. Particularly, DRD2 is the gene common to both the list of genetic variations associated with substance addiction and the components of the brain neuronal signaling network involved in substance addiction. IPA identified multiple links between components of these 2 lists of genes. ERK1/2 was directly connected to DRD1 and indirectly connected to RAC1, FOS, ERK, Creb, PI3K, BDNF and Pka in the signaling network in neuronal adaptation/plasticity in substance addiction (i.e., reward circuit). All the commonly shared immune response genes for the three addiction phenotypes, including TLR6, CXCL5, CXCL3, CRHR1 and CCL21, were indirectly linked to NFkB in the reward circuit. Gene CCL21 was also indirectly linked to Akt and ERK in the reward circuit.

## Discussion

One of the most challenging areas of oncologic medicine is the management and treatment of severe, chronic pain that arises from cancer therapies, including surgery, chemotherapy, and radiation, as well as cancer itself. Opioids remain the drugs of choice for cancer pain management [54], however, the use of opioids for treatment of chronic pain in cancer patients remains debatable. An increasing concern is the potential rise in aberrant drug-taking behaviors of cancer patients undergoing treatment for chronic pain [3, 55]. Given that addictions to alcohol and tobacco are known risk factors for cancer, exploring genetic markers of risk for these addiction phenotypes in cancer patients may help in risk stratification. Indeed, studies have begun to show that genetic vulnerability to different substances of addiction may partly overlap [56]. The primary aims of this study were to determine whether there exists a genetic basis to the relationship between smoking, alcohol, and opioid addiction, and to identify candidate genes associated with the three phenotypes for further study.

We used IPA, a bioinformatics tool, to identify commonly shared genes for alcohol, smoking, and opioid addiction. Of the 20 genes commonly shared across the alcohol, smoking and opioid addiction phenotypes, extracellular-signal-regulated kinases 1 and 2 (ERK1/2) was found to have the most interconnections across all three addiction networks as indicated by the number of edges (biological interactions; Table 4). Recent studies suggest the relevance of ERK pathway in drug addiction. Several studies have cited the role of ERK in brain’s response to drugs of abuse [57–59]. Specifically, Valjent et al. [59] demonstrated that multiple drugs of abuse increased activation of ERK1/2. Molecular mechanisms underlying ERK1/2 activation by drugs of abuse and the role of ERK1/2 signaling in long-term neuronal plasticity in the striatum may provide novel targets for therapeutic intervention in addiction [60]. Moreover, studies exploiting ERK activation for cancer therapy have been promising, including the use of MEK inhibitors to block ERK activation in acute lymphoblastic leukemia for instance [61]. Future studies are needed to assess the potential clinical relevance of ERK1/2 for addiction, e.g., to genotype ERK1/2 and stratify patients for prompt intervention, or to determine appropriate dosage of opioid analgesics to patients with specific genotypes.

Of note, the identified shared genes for the three addiction phenotypes are involved in immune response. This is consistent with recent research that implicates immune signaling in drug addiction. Dafney et al. demonstrated that certain immunosuppressive treatments controlled morphine withdrawal in rats [62, 63]. More recent studies demonstrated that blocking pro-inflammatory glial activation could block the elevation of dopamine induced by opioid receptor activity [64, 65]. Hutchinson et al. have also found evidence that toll-like receptors (TLRs), a class of innate immune receptors, interact with opioids and glial cells, contributing to opioid reward behaviors [65]. Our recent studies also showed that cytokine genes are implicated in pain, depressed mood, and fatigue in cancer patients [66–68], and these cytokines may serve as biomarkers of risk for persistent pain in cancer patients.

Furthermore, it is also speculated that synaptic plasticity induced by substances of abuse in the neuronal circuits of reward may underlie behavioral changes that characterize addiction. Importantly, NF-kappa B may be the link between inflammation and neuronal/synaptic plasticity involved in behavioral changes in addiction, as we have shown that all the commonly shared immune response genes of three addiction phenotypes were linked to NF-kappa B in the reward circuit (Fig. 5). NF-kappa B is one of several transcription factors present at the synapse, and it is activated by brain-specific activators such as glutamate (via AMPA/KA and NMDA receptors) and neurotrophins [69]. To date, there are currently no pharmacotherapies for drug addiction targeting immune signaling.

Our results also showed the top canonical pathways associated with all the 56 focus genes of three addiction phenotypes were: 1) calcium signaling, 2) GPCR signaling, 3) cAMP-mediated signaling, 4) GABA receptor signaling, and 5) Gαi signaling. These pathways have been confirmed to be associated with substance addiction in the literature [70–74]. They are the post-receptor signaling pathways for the glutaminergic, dopaiminergic and GABAergic neurons involved in the “reward circuitry” in mammalian brains [75]. Whether these pathways can be used as targets for drug addiction therapy needs to be explored. Our approach of identifying genetic variations associated with addiction to multiple substances and linking to known the neural signaling network involved in substance addiction in the brain has clarified the functional significance of many of the genetic associations to substance addiction. This bioinformatics approach has also identified signaling pathways that may be targeted by drugs. Promising research has shown that allosteric modulators of GPCRs can be used to treat addiction by altering the affinity of the GPCR to its ligand or impacting its downstream signaling responses [72]. Other studies have also suggested positive allosteric modulation of GABA_B_ as a therapeutic strategy for treatment of addiction [71, 76].

Among the limitations of this study is that edges are simplified in the IPA designates only a single edge between each pair of molecules in a network regardless of the number of interactions the two molecules share. Furthermore, this bioinformatics analysis is hypothesis-generating, and the findings must be further investigated and validated experimentally.

## Conclusions

Studying smoking, alcohol, and opioid addiction phenotypes in conjunction allowed us to identify molecules and pathways involved in multiple types of drug addiction. IPA is able to use large-scale information to produce comprehensive networks of genes and underlying biological pathways implicated in a phenotype [19]. Most of the current literature on addiction genes focuses on genes specific to each type of addiction, while in this study we studied genes relating to multiple addiction phenotypes. Our findings show immune signaling and ERK1/2 as novel genetic markers for multiple addiction phenotypes including alcohol, smoking and opioid addiction. Future studies are needed to validate our findings in large cohorts of patients.

## Additional files

Additional file 1: Figure S1. Network generated using 25 focus genes for alcohol addiction (p-score = 16). **Figure S2.** Network generated using 27 focus genes for nicotine addiction (p-score = 31). **Figure S3.** Network generated using 15 focus genes for opioid addiction (p-score = 10).
