# Supplementary material for: Gene network analysis shows immune-signaling and ERK1/2 as novel genetic markers for multiple addiction phenotypes: alcohol, smoking and opioid addiction
Source: BMC Syst Biol. 2015 Jun 5;9:25. doi: 10.1186/s12918-015-0167-x (PMC4456775; doi:10.1186/s12918-015-0167-x)
Supplement: Additional file 1: Figure S1. — Network generated using 25 focus genes for alcohol addiction (p-score = 16). Figure S2. Network generated using 27 focus genes for nicotine addiction (p-score = 31). Figure S3. Network generated using 15 focus genes for opioid addiction (p-score = 10). [file 12918_2015_167_MOESM1_ESM.docx]

**Additional file 1**


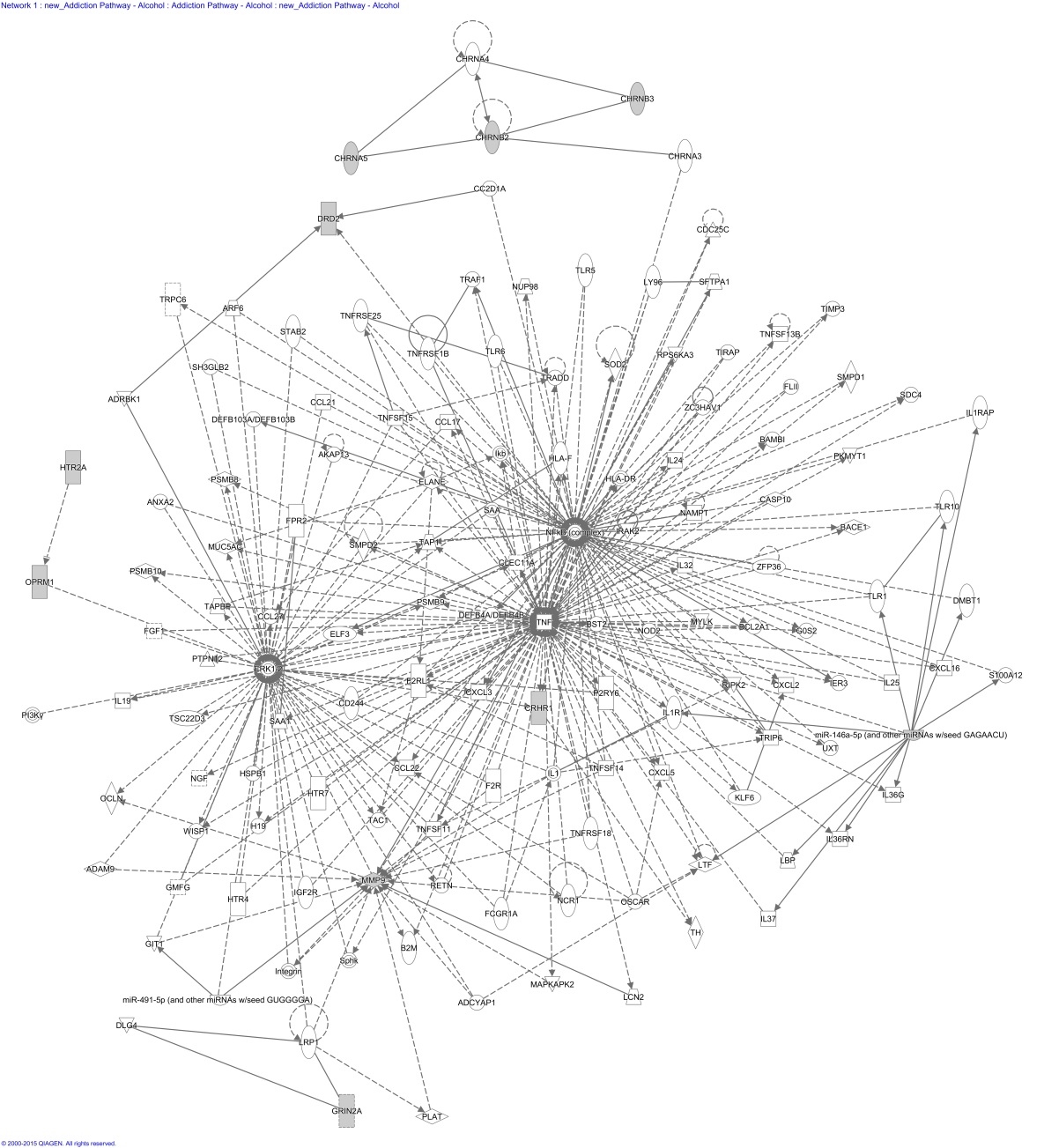


Figure S1: Network generated using 25 focus genes for alcohol addiction (p-score = 16)

Focus genes associated with alcohol addiction


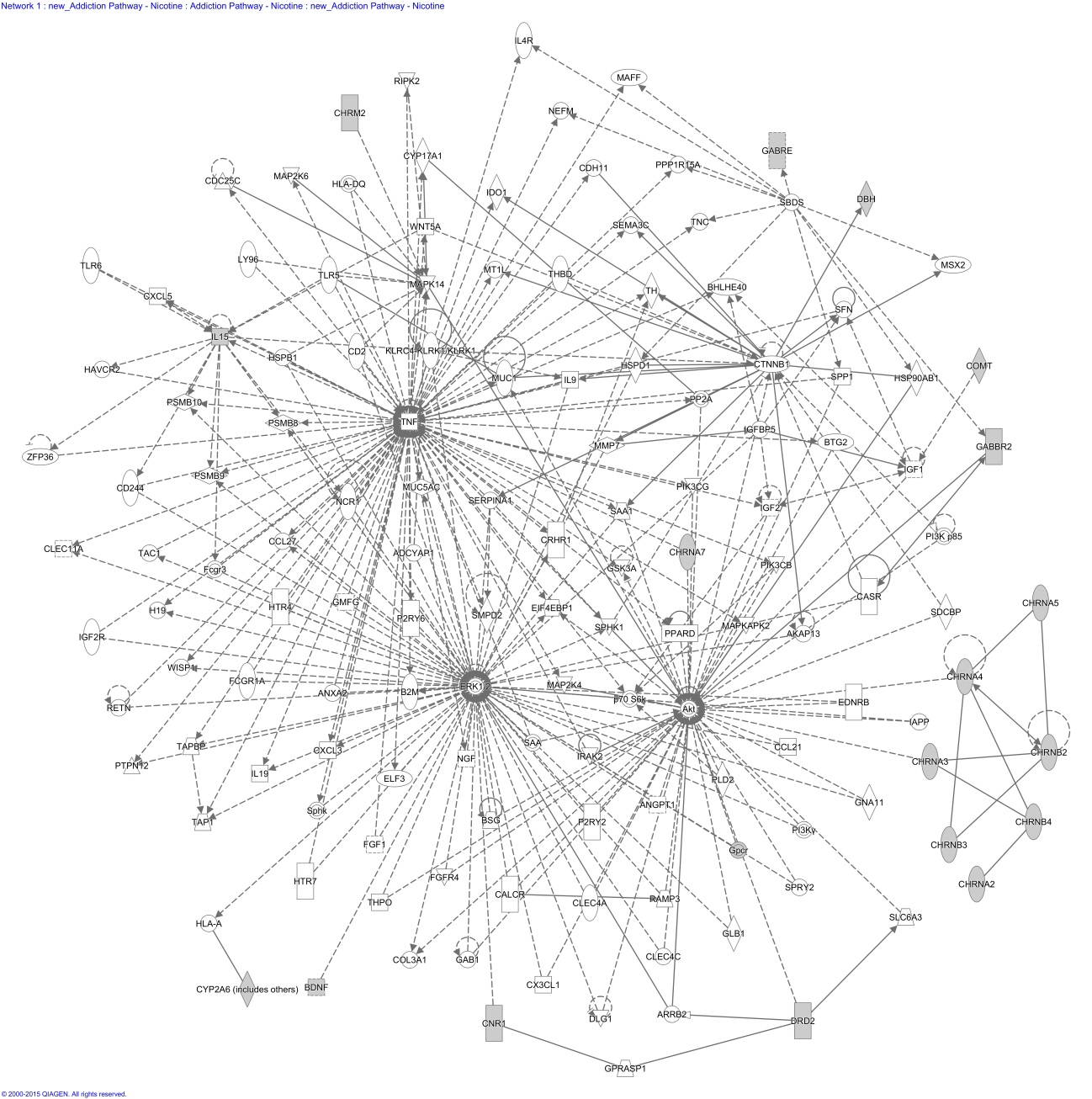


Figure S2: Network generated using 27 focus genes for nicotine addiction (p-score = 31)

Focus genes associated with nicotine addiction


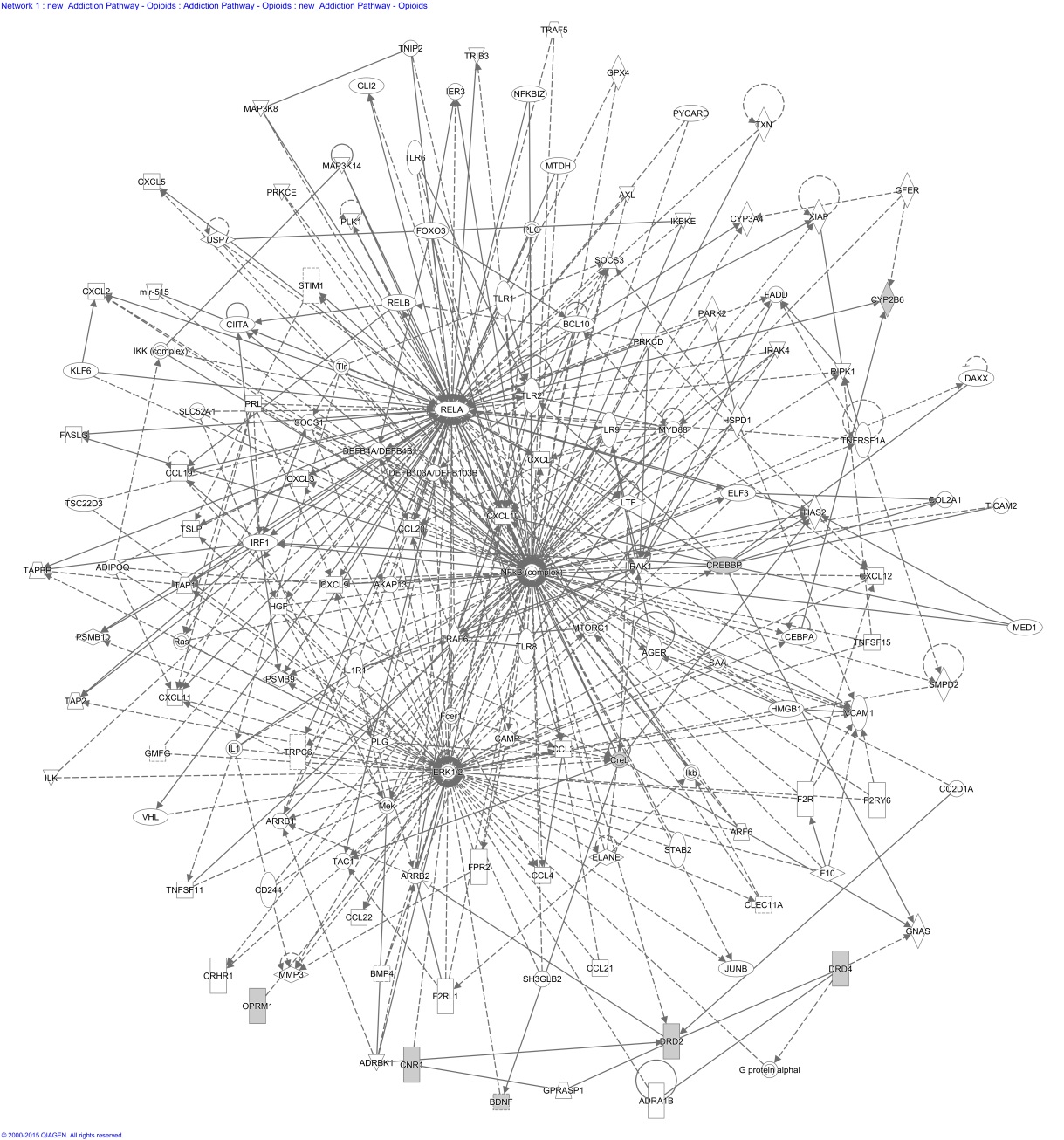


Figure S3: Network generated using 15 focus genes for opioid addiction (p-score = 10)

Focus genes associated with opioid addiction
